# Sox2 and βIII-Tubulin as Biomarkers of Drug Resistance in Poorly Differentiated Sinonasal Carcinomas

**DOI:** 10.3390/jpm13101504

**Published:** 2023-10-18

**Authors:** Luis López, Laura Fernández-Vañes, Virginia N. Cabal, Rocío García-Marín, Laura Suárez-Fernández, Helena Codina-Martínez, Sara L. Lorenzo-Guerra, Blanca Vivanco, Verónica Blanco-Lorenzo, José L. Llorente, Fernando López, Mario A. Hermsen

**Affiliations:** 1 Department of Otolaryngology, Hospital Universitario Central de Asturias, 33011 Oviedo, Spain; luislf1996@gmail.com (L.L.); laufva@gmail.com (L.F.-V.); jllorentep@uniovi.es (J.L.L.); 2 Department of Head and Neck Cancer, Instituto de Investigación Sanitaria del Principado de Asturias (ISPA), 33011 Oviedo, Spain; vircabal@hotmail.com (V.N.C.); rociogm220879@hotmail.com (R.G.-M.); suarezflaura@gmail.com (L.S.-F.); helenacm14@gmail.com (H.C.-M.); saralgcv96@gmail.com (S.L.L.-G.); mhermsen@hca.es (M.A.H.); 3 Department of Pathology, Hospital Universitario Central de Asturias, 33011 Oviedo, Spain; vivancoblanca@uniovi.es (B.V.); veronica.blanco@sespa.es (V.B.-L.)

**Keywords:** poorly differentiated sinonasal carcinomas (PDCs), sinonasal undifferentiated carcinoma (SNUC), olfactory neuroblastoma (ONB), sinonasal neuroendocrine carcinoma (SNEC), SOX2, βIII-tubulin, prognostic biomarker

## Abstract

Poorly differentiated sinonasal carcinomas (PDCs) are tumors that have a poor prognosis despite advances in classical treatment. Predictive and prognostic markers and new personalized treatments could improve the oncological outcomes of patients. In this study, we analyzed SOX2 and βIII-tubulin as biomarkers that could have prognostic and therapeutic impacts on these tumors. The cohort included 57 cases of PDCs: 36 sinonasal undifferentiated carcinoma (SNUC) cases, 13 olfactory neuroblastoma (ONB) cases, and 8 sinonasal neuroendocrine carcinoma (SNEC) cases. Clinical follow-up data were available for 26 of these cases. Sox2 expression was detected using immunohistochemistry in 6 (75%) SNEC cases, 19 (53%) SNUC cases, and 6 (46%) ONB cases. The absence of Sox2 staining correlated with a higher rate of recurrence (*p* = 0.015), especially distant recurrence. The majority of cases showed βIII-tubulin expression, with strong positivity in 85%, 75%, and 64% of SNEC, ONB, and SNUC cases, respectively. Tumors with stronger βIII-tubulin expression demonstrated longer disease-free survival than those with no expression or low expression (*p* = 0.049). Sox2 and βIII-tubulin expression is common in poorly differentiated sinonasal tumors and has prognostic and therapeutic utility.

## 1. Introduction

Poorly differentiated sinonasal carcinomas (PDCs) are a heterogeneous group of neoplasms that can be divided into several subtypes. Tumors with a neuroectodermal origin, like olfactory neuroblastoma (ONB), and those with an epithelial origin, like sinonasal neuroendocrine carcinomas (SNECs) and sinonasal undifferentiated carcinomas (SNUCs), are included in the PDC group [1,2]. However, the classification of PDCs is often difficult due to overlapping histological features [3]; for example, the term “olfactory carcinoma” was recently created in order to classify a high-grade tumor that presents both neuroendocrine and epithelial characteristics [4]. In addition, the fifth edition of the WHO classification considers SNEC as equal to other poorly differentiated neuroendocrine tumors located in other anatomic regions [3,5]. Furthermore, new tumor subtypes are defined by characteristic molecular genetic changes affecting IDH2, NUT, or genes in the SWI/SNF pathway in SNUC, which may also have therapeutic implications [3,6,7]. Investigations into molecular profiling continue to improve and refine classification and discover optimal therapeutical approaches for each of them [8,9].

In clinical terms, PDCs have moderate-to-poor outcomes owing to aggressive tumor biology. Although multimodal treatment is recognized as the most effective, patients treated with surgery, chemotherapy (CT), and radiotherapy (RT) frequently experience tumor progression and poor survival [10,11,12,13]. PDCs arise in the tops of the nasal cavities, with anatomical and radiological evidence supporting their origin in the olfactory epithelium. A recent study yielded the first molecular evidence demonstrating that ONB derives from the globose stem cells of the olfactory epithelium [4]. These progenitor cells are located in the basal layer, and during their differentiation process, the expression patterns of various proteins change [4,14]. Aside from their roles as cancer-related genes, both SOX2 and TUBB3, encoding Sox2 (Sex-determining region Y-box 2) and βIII-tubulin, respectively, play a role in the differentiation from basal cells to committed progenitor cells and, finally, adult cells [4,15].

Sox2 is a transcription factor expressed in pluripotent cells during embryonic development as well as in somatic stem cells in adult tissue, including the olfactory epithelium [15,16,17]. It regulates, in association with other factors, the reprogramming of terminally differentiated somatic cells back to a pluripotent stem cell state when co-expressed with other embryonic stem cell factors [18,19,20]. In addition, SOX2 also plays an important role as an oncogene involved in the growth capacity of tumor stem cells. The loss of expression of Sox2 significantly reduces the ability of tumor cells to grow and expand. Conversely, the overexpression of Sox2 results in a greater capacity for tumor progression [21]. Sox2 expression has been shown in several tumor types, such as lung, esophagus, breast, skin, prostate, and ovarian tumors, as well as in squamous carcinomas of various localizations and sarcomas [21,22,23,24,25,26,27,28,29]. In the head and neck region, Sox2 expression and gene amplification have been identified as common events [30,31]. Among the tumors that originate in the sinonasal cavities, the amplification and/or overexpression of Sox2 was demonstrated in squamous sinonasal carcinoma (SNSCC), SNUC, adenoid cystic carcinomas (ACCs), and ITAC [29,32]. The relation of Sox2 expression (and SOX2 gene copy number gain) with clinical outcomes is still a matter of debate, suggesting distinct roles for SOX2 depending on the tumor localization and histology. However, in a recent meta-analysis [33], a high expression level of Sox2 was significantly associated with poor outcomes in patients with solid tumors, which is consistent with a role as a transcription factor that induces stemness [34]. Sox2 expression is also associated with resistance to chemotherapy through a plethora of mechanisms and, as such, is a promising target for anticancer therapy [17,34].

Microtubules are multifunctional cytoskeletal proteins involved in critical cellular processes, such as mitosis, cell motility, and intracellular transport. Microtubules are composed of polymers of α- and β-tubulin heterodimers. Class III β-tubulin (βIII-tubulin) is one kind of β-tubulin and is typically expressed in cells of neuronal origin [15]. It contributes to the formation of dynamic microtubules essential for neurite formation and maintenance. An abnormal βIII-tubulin content may also be relevant in the development of chromosomal instability, as it is believed to contribute to centrosomal amplification, multipolar spindle poles, and the missegregation of chromosomes [35]. This is why several lines of evidence suggest that βIII-tubulin plays a role in tumor development. The overexpression of βIII-tubulin has been associated with adverse clinical features and poor outcomes in many epithelial tumor types, such as non-small-cell lung, gastric, breast, colon, bladder, prostate, and ovarian cancers [36,37,38,39,40,41,42]. This is also true for head and neck cancer, where βIII-tubulin overexpression has been linked to a shortened survival of patients [43,44]. Nevertheless, some studies have found a high prevalence of βIII-tubulin expression without an association with clinical outcomes [45]. 

The effects of βIII-tubulin overexpression on taxane and other anti-microtubule agent (MTA)-based chemotherapies have been investigated in various tumors [46]. A recent study by Topcagic et al. demonstrated an association between high βIII-tubulin expression and resistance to vincristine in ONB [47]. This could be related to the fact that most anti-microtubule agents act by binding to the β-subunit [48]. New strategies to target βIII-tubulin that comprise inhibiting its expression using small molecules and using MTAs that evade drug efflux pumps or bind the colchicine site of tubulin instead of the taxane site are under investigation [35]. 

To better understand their role as prognostic or therapy-response-predictive factors in PDCs, we studied Sox2 and βIII-tubulin protein expression in a series of 57 tumors of three different histologies, namely, SNUC, ONB, and SNEC, and we correlated the results with clinicopathological and follow-up data. In addition, we discuss the role of these markers in the response to CT.

## 2. Materials and Methods

### 2.1. Patients and Methods

A total of 57 cases (36 SNUC cases, 13 ONB cases, and 8 SNEC cases) treated between 1998 and 2017 were included in our study after receiving informed consent and approval by the Ethics Committee of the Hospital Universitario Central de Asturias Hospital. This study was conducted according to the guidelines of the Declaration of Helsinki. Tumors of similar lineages such as NUT carcinoma and SMARCA4-deficient and SMARCB1-deficient carcinoma were excluded from this study. All patients had a single primary tumor and had received no treatment prior to surgery. The clinical features of the three groups of patients are shown in Table 1. Most tumors were located in the ethmoid sinus (89%). According to the 8th edition of the International Union Against Cancer TNM classification [48], 75% of the cases were in the advanced stages (stages III and IV). No patient had distant metastases at the time of diagnosis. Ninety-five percent (54/57) of the patients received postoperative RT. Follow-up information was obtained for 26 patients until the last occurrence for patients still alive, until the time of death, or until the time of lost contact. The median follow-up time for the entire group was 16 months (range: 1–172).

### 2.2. Histological Classification of Sinonasal Tumors

**Figure 1:**
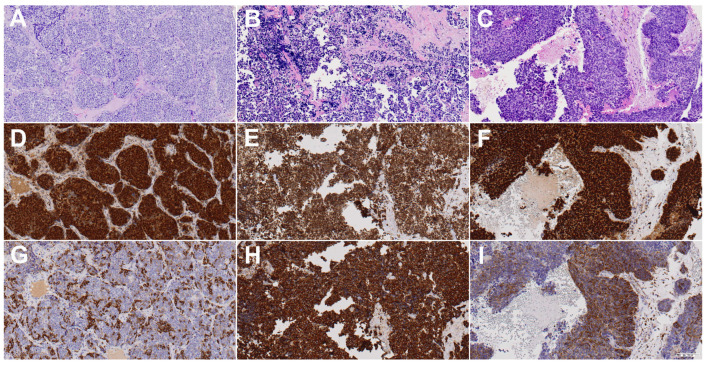
H&E, Sox2, and βIII-tubulin staining images of one representative case each of sinonasal undifferentiated carcinoma (**A**,**D**,**G**), olfactory neuroblastoma (**B**,**E**,**H**), and sinonasal neuroendocrine carcinoma (**C**,**F**,**I**). Sox2 was positive in all three cases, while βIII-tubulin was determined to be moderate in (**G**,**I**) and strong in (**H**). All images show 20× magnification.

As the cases were collected over a long period of time and at different institutions, the initial diagnoses were reviewed by two experienced pathologists (BV and VBL). All available slides were reviewed in each case, and tumors were diagnosed according to the diagnostic criteria of the 5th edition of the WHO classification [49]. Additional immunohistochemical staining of cytokeratin 5/6, p40, p16, synaptophysin, chromogranin, NUT, SMARCB1, and SMARCA4 were applied when necessary. Cases positive for p16 were further tested for HPV using DNA-PCR (see below). As a result of these diagnostic analyses, none of the cases were reclassified as NUT carcinoma or SMARCB1-deficient or SMARCA4-deficient carcinoma, and all tumors with diffuse and nuclear p16 staining were found to be HPV-negative. The immunohistochemical and genetic diagnostic results are included in Table 2. Representative H&E staining images of the three tumor types (SNUC, ONB, and SNEC) are shown in Figure 1.

### 2.3. Immunohistochemistry

Tissue microarray (TMA) blocks were prepared from formalin-fixed, paraffin-embedded tumor tissues using a Beecher Tissue Microarray (Beecher Instruments, Silver Spring, MD, USA). The TMA blocks contained three 1 mm cores from different areas of each tumor. Normal sinonasal mucosa samples were included in each block as an internal control. Tumors not included in the TMAs were stained separately. Immunohistochemistry was performed with an automated staining workstation (Dako Autostainer Plus; DakoCytomation, Glostrup, Denmark) with antigen retrieval using EnVision FLEX + Mouse (DakoCytomation, Glostrup, Denmark) for 20 min. 

The following antibodies were applied when necessary for tumor classification: mouse anti-Cytokeratin clone AE1/AE3, mouse anti-Synaptophysin clone SY38, mouse anti-Chromogranin A clone DAK-A3, mouse anti-CK 5/6 clone D5/16 B4, mouse anti-p63 clone DAK-p63 (DAKO, Glostrup, Denmark), mouse anti-p40 clone BC-28, mouse CINtec^®^ anti-p16 clone E6H4 (Roche, Mannheim, Germany), rabbit anti-BRG1 clone ab70558 (Abcam, Cambridge, UK), rabbit anti-SMARCB1/BAF47 clone D8M1X, and rabbit anti-NUT clone C52B1 (Cell Signaling Technology, MA, USA). 

The antibodies for the Sox2 and βIII-tubulin expression analysis were rabbit anti-Sox2 AB5603 (Millipore Ibérica SA Madrid, Spain) and mouse anti-Btub-III G712A (Promega, Madison, WI, USA), both applied at a dilution of 1:1000, with an antigen retrieval incubation time of 20 min at 95 °C in a citrate buffer (pH 9.0). Negative controls were prepared by omitting the primary antibody. The immunohistochemical results were evaluated independently by three observers (L.F-V., B.V., and V.B.L.) without knowledge of the clinicopathological outcomes of the patients. In samples where there were discrepancies, they were solved afterwards by looking together using a multi-head microscope, and a consensus was reached. Sox2 expression was scored as positive when >5% of cells showed nuclear expression [50,51,52]. βIII-tubulin was scored by intensity (0, 1, 2, or 3) and percentage of positivity (1 as <30%, 2 as 30–70%, and 3 as >70%); the multiplication factor of both scores produced negative staining when 0, weak staining when 1–2, moderate staining when 3–4, and strong staining when 6–9 [40]. Representative Sox2 and βIII-tubulin staining images of the three tumor types (SNUC, ONB, and SNEC) are shown in Figure 1.

### 2.4. HPV DNA and IDH2 Mutation Detection

The quality of the extracted DNA was checked via PCR amplification of β-globin (forward primer 5′-ACACAACTTGTGTGTTCACTAGC-3′ and reverse primer 5′-CAAACTTCATCCACGTTCACC-3′). PCR with MY11/GP6+ primers (site-directed L1 fragment of HPV) was performed on 9 cases of p16-positive SNUC in order to detect a broad spectrum of HPV genotypes [53]. Briefly, the PCR was performed in 25 µL of a reaction mixture containing 1x PCR buffer, 2 mmol/L MgCl2, 50 µmol/L of each deoxynucleoside, 0.5 µmol/L of sense and antisense primers, 10 µL of a DNA sample, and 1 U of Taq DNA polymerase (Promega Biotech Iberica S.L., Madrid, Spain) with a thermal profile of 35 cycles of denaturation at 94 °C for 30 sec, annealing at 55 °C for 30 sec, and extension at 72 °C for 1 min, with an initial denaturation at 94 °C for 5 min and a final extension at 72 °C for 10 min. Amplified DNA fragments of approximately 200 bp were identified via electrophoresis in 1.5% agarose gel with ethidium bromide.

IDH2 mutations in SNUC identified via Sanger PCR sequencing and immunohistochemistry with the multi-specific antibody mouse anti-IDH1/2 mutant R132/172 clone MsMab-1 at a 1:100 dilution (Millipore, Darmstadt, Germany) were studied previously [53]. 

### 2.5. Statistical Analysis

A univariate analysis using Pearson χ2 and Fisher’s exact tests was used for comparisons between categorical variables using SPSS 19.0 software for Windows (SPSS Inc., Chicago, IL, USA). For the time-to-event analysis, Kaplan–Meier curves were plotted. Differences between survival times were analyzed using the log-rank method. Values of *p* ≤ 0.05 were considered statistically significant.

## 3. Results

### 3.1. Clinicopathological Data and Follow-Up

Follow-up information, including local recurrence and patient status, was available for 26 cases (Table 1). Local recurrence was found in 5/16 (31%) SNUC patients, 3/7 (43%) ONB patients, and 2/3 (67%) SNEC patients. Distant metastasis occurred in 5/16 (31%) SNUC patients, 3/7 (43%) ONB patients, and 3/3 (100%) SNEC patients. Six patients (three SNUC patients, two SNEC patients, and one ONB patient) presented local recurrence as well as distant metastasis. 

**Figure 2:**
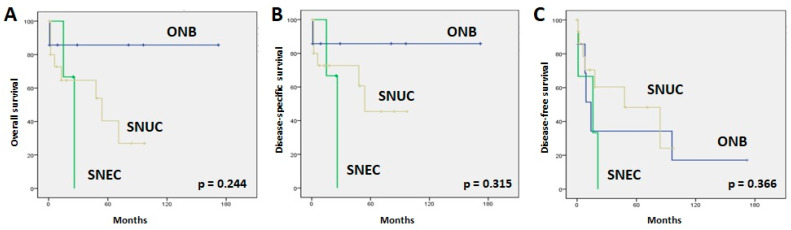
Kaplan–Meier overall (**A**), disease-specific (**B**), and disease-free (**C**) survival curves according to tumor type.

The 5-year overall survival (5-yr OS) rates were 86%, 40%, and 0% for all ONB, SNUC, and SNEC cancer patients, respectively (Figure 2). Similar figures were observed for the 5-year disease-specific survival (5-yr DSS) (Figure 2). The median disease-free times were 14 months (95% CI: 7–21) for ONB, 16 months (95% CI: 1–40) for SNEC, and 48 months (95% CI: 1–104) for SNUC. The 5-year disease-free survival (5-yr DFS) rates were 34%, 48%, and 0% for all ONB, SNUC, and SNEC patients, respectively (Figure 2). The main causes of death were local recurrences and distant metastases. At the time of writing, a total of 15 of 26 patients (57%) remained alive without disease. We observed no significant relations between OS, DSS, or DFS and clinical parameters such as gender, age, location, or tumor stage.

### 3.2. SOX2 Expression

**Figure 3:**
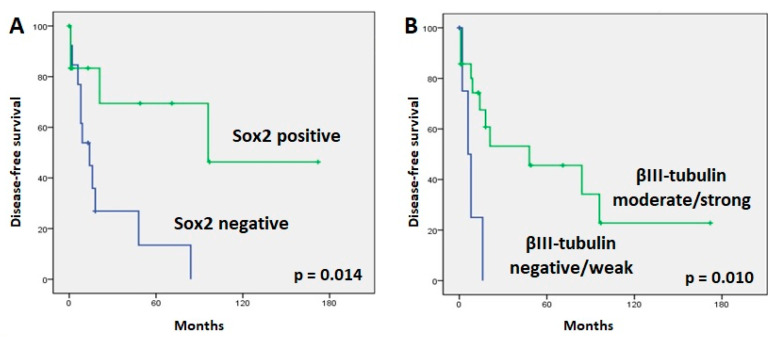
Kaplan–Meier plots of disease-free survival curves according to Sox2 (**A**) and βIII-tubulin (**B**) expression.

In normal sinonasal mucosa, nuclear Sox2 staining was found in stem cells in the basal layer and especially in sustentacular cells in a layer closer to the surface of the mucosa, thus creating a characteristic “sandwich” staining pattern. Examples of tumoral positive staining for Sox2 are shown in Figure 1. SNEC showed more positive cases than SNUC and ONB, at 6/8 (75%), 19/36 (53%), and 6/13 (46%), respectively (Table 3), but the difference was not significant (Fisher exact chi square test, *p* = 0.414). When all histological subtypes were analyzed together, we found that the absence of Sox2 expression correlated significantly with a higher rate of relapse (11/13 patients (85%) without expression versus 4/13 patients (31%) with Sox2 expression; Fisher exact chi square test, *p* = 0.015). Accordingly, the 5-yr DFS was higher in patients with Sox2 expression (68%) than in patients without Sox2 expression (13%) (log rank 6.022, *p* = 0.014) (Figure 3). In addition, patients without Sox2 expression tended to develop more distant metastases (9/13 patients (70%) without expression versus 2/13 patients (15%) with Sox2 expression; Fisher exact chi square test, *p* = 0.015). Sox2 expression showed no correlation with local recurrences (10/13 patients (77%) with expression versus 6/13 patients (46%) without Sox2 expression; Fisher exact chi square test, *p* = 0.113). No correlations were observed with other clinicopathological parameters, including localization and stage. We did not observe a significant correlation between Sox2 expression levels and OS or DSS (respectively, *p* = 0.851 and *p* = 0.720). A survival analysis for each tumor subtype was not feasible due to the low number of cases with follow-up information.

### 3.3. βIII-Tubulin Expression 

The immunohistochemical staining of βIII-tubulin was exclusively positive in mature and immature neurons present in normal olfactory mucosa but not in sinonasal respiratory mucosa. Examples of tumoral staining for βIII-tubulin are shown in Figure 1. Positive staining for βIII-tubulin was detected in 52/57 (91%) cases (Table 3). βIII-tubulin staining in ONB was considered strong in 11/13 (85%) cases and moderate in 1/13 (8%) cases. Strong, moderate, and weak βIII-tubulin staining were observed in 6/8 (75%), 1/8 (12%), and 1/8 (12%) SNEC samples. In SNUC, staining for βIII-tubulin was rated strong in 23/36 (64%) samples, moderate in 3/36 (8%) samples, and weak in 6/36 (17%) samples (Table 3). Thirty cases (58%) with positive staining for βIII-tubulin also showed Sox2 overexpression.

Cases with moderate/strong βIII-tubulin staining demonstrated a significant association with longer DFS tumors (*p* = 0.010) (Figure 3). However, βIII-tubulin expression did not correlate with the development of a recurrence or a distant metastasis during follow-up. Advanced-stage tumors tended to have more frequent βIII-tubulin expression (32/33 advanced-stage patients (97%) showed βIII-tubulin expression versus 20/24 early-stage patients (93%); Fisher exact chi square test, *p* = 0.094). There were no significant variations in OS or DSS regarding βIII-tubulin expression. No correlation was found when analyzing each tumor type separately.

## 4. Discussion

Sinonasal tumors are among the most difficult malignancies to treat. As we are beginning to know more of the molecular pathways involved, a new range of possibilities for adjuvant treatments and conservative protocols is coming into view. In the present study, we analyzed the expression of βIII-tubulin and Sox2 as potential biomarkers for prognosis and treatment.

Sox2 is a critical transcription regulator and has been postulated as a driver oncogene [54,55]. Although epigenetic silencing has been observed [56], several publications have reported overexpression of Sox2, supporting its role as an oncogene [57,58]. Our findings demonstrate that Sox2 expression is a frequent event in PDC tumorigenesis (54% of samples). Schröck et al. [32] assessed SOX2 gene amplification and protein expression using fluorescence in situ hybridization and immunohistochemistry, respectively, in 159 sinonasal tumors, including 59 squamous cell carcinomas (SCCs), 18 SNUCs, 10 carcinomas associated with an inverted papilloma (INVCs), 19 adenocarcinomas, and 13 adenoid cystic carcinomas. They found SOX2 amplifications and its associated overexpression in more than a third of all SCCs, SNUCs, and INVCs. Hence, Sox2 expression appears to be a frequent event in sinonasal tumors. Most studies on SOX2 have concluded that protein overexpression is significantly correlated with tumor recurrence and poor prognosis [59,60], which is compatible with its function as a transcription factor linked to stemness [34]. However, the role of SOX2 is still controversial, as there have been other studies that did not reach the same conclusions [22,29]. Specifically in sinonasal tumors, the results are less convincing with respect to prognosis; a significant correlation between Sox2 expression levels and tumor recurrence was not observed [29,32]. In contrast, our results showed that positive expression of Sox2 was correlated with a lower rate of distant metastasis and better DFS. These results are similar to those reported by Bayo et al. in head and neck squamous cell carcinoma. They hypothesized that Sox2 inhibits tumor cell motility in cancer cells and that low Sox2 expression serves as a prognosticator to identify patients at high risk of relapse [61]. It is also possible that these contradictory results are related to methodological differences in Sox2 immunoscoring; three different approaches have been used in the literature to evaluate its staining [29]. Another explanation for these contradictory results may be that SOX2 expression is an early tumor-initiating event and is therefore important for tumor development but is not involved in conveying an aggressive or metastasizing phenotype [30].

βIII-tubulin upregulation is a common feature that occurs with different frequencies in almost every type of cancer [62]. To our knowledge, this is the first study of βIII-tubulin expression in PDC. A high expression of βIII-tubulin was observed in most of our samples (91%). Nienstedt et al. studied the expression of βIII-tubulin in samples from 667 cancers of the oral cavity, oro- and hypopharynx, and larynx, and over 90% of the analyzed cancers showed cytoplasmic βIII-tubulin expression [45]. Thus, PDC shares this characteristic with head and neck tumors. βIII-tubulin may have an important role in oncogenesis due to its function as a part of the microtubules. However, the role of βIII-tubulin as a prognostic marker is not well established. While increased βIII-tubulin has been associated with aggressiveness, resistance to chemotherapy, and poor clinical outcomes in some solid tumors [38,44,63,64], other studies have failed to identify an association between βIII-tubulin expression and clinicopathological data [37,45]. In our study, no significant correlations between βIII-tubulin and clinicopathological parameters were observed, in spite of high expression levels. We did observe a significant association between higher βIII-tubulin expression and longer DFS, similar to Lobert et al. [65], who reported a trend of increased βIII-tubulin mRNA in cases with longer DFS. The controversial prognostic value of βIII-tubulin has been pointed out by other authors, suggesting that βIII-tubulin is a pure prognostic biomarker only when its expression is conditioned by a toxic microenvironment. However, when its expression is driven by other factors, the role of βIII-tubulin can be neutral or even opposite, acting as a marker of a more differentiated and less aggressive disease [66]. On the other hand, the discrepant conclusions between studies could be due to variances in methodology, study design, and patient selection or the small cohort sizes of the studies.

CT is often used to treat PDC. Both βIII-tubulin and SOX2 have been implicated in mechanisms of chemoresistance [44,67]. High levels of βIII-tubulin have been associated with resistance to anti-microtubule agents, so testing for this marker could be helpful to identify patients who might benefit the most from these treatments [47,64,68]. Nevertheless, some authors state that βIII-tubulin overexpression does not affect anti-microtubule-based CT [69]. Moreover, βIII-tubulin expression may present different biological characteristics in different tumors. Our patient data did not include CT information, and further studies are needed to clarify the role of this marker in chemotherapeutic resistance in PDC. Also, SOX2 has been linked with chemoresistance. There are a plethora of different mechanisms contributing to SOX2-induced resistance to therapy as part of its role in inducing stemness [34,67]. Moreover, SOX2 can induce protective autophagy and repress apoptosis, which are also linked to drug resistance. SOX2 is, therefore, an attractive therapeutic target to overcome drug resistance. Because SOX2 is still an undruggable transcription factor, the discovery of selective inhibitors is very limited, although some SOX2-targeting approaches have been undertaken [67]. In addition to SOX2, other stemness-targeting factors [20] have been associated with better responses to CT [70].

Another candidate treatment option for PDC is immunotherapy, although few studies have studied immune checkpoint inhibitors in these tumors. Recent immunotherapeutic biomarker studies have reported CD8+ TILs in up to 20% of ONBs, which were associated with a more favorable prognosis [71,72,73]. PD-L1 expression occurred in 14–20% of ONBs. CD8+ TILs and PD-L1 expression have also been observed in 17% and 33% of SNECs, respectively, and in 33% and 16% of SNUCs, respectively [73]. Other biomarkers include the tumor mutation burden and microsatellite instability, but few data on PDC are available. Using an immunohistochemical analysis of PMS2, MLH1, MSH2, and MSH6, Villanueva et al. [73] identified MSI in 2/25 SNUCs, 2/6 SNECs, and 0/14 ONBs, while in a large study of different sinonasal cancer types Hieggelke et al. [74] found MSI in only 4/125 squamous cell carcinomas but not in SNUCs or SNECs. These first studies indicate that a considerable subset of PDC patients could benefit from immunotherapy.

Most PDCs arise in the top of the nasal fossa, near the area of the olfactory groove, showing variable degrees of neuroendocrine differentiation [75]. Due to these features, we hypothesize that they have a shared origin with different hits during their evolution that would mark the differences in their histopathology and clinical behavior. Following injury, quiescent olfactory stem cells rapidly shift to activated, transient states that are unique to regeneration and are tailored to meet the demands of injury-induced repair. Multiple cell fates, including renewed stem cells, committed differentiating progenitors, and cancer stem cells, have been specified during this early window of activation [4,14,76]. Human neural stem/progenitor cells derived from the olfactory epithelium express the protein markers Sox2 and βIII-tubulin [77]. We found frequent Sox2 expression in a series of PDCs, possibly in line with the role of Sox2 in cancer stem cells. SOX2 cross-talks with multiple signaling pathways. The EGFR/MAPK/PI3K-mTOR-AKT and Wnt/β-catenin signaling pathways are implicated in some sinonasal carcinomas [2,78]. Sox2 overexpression promotes cell proliferation and migration by activating these pathways, which at the same time can affect Sox2 expression [67]. Sox2 also induces the expression of some epithelium-specific genes, including βIII-tubulin [79]. In our study, 30/57 samples were Sox2+/βIII-tubulin+. Over 90% of our analyzed cancers showed cytoplasmic βIII-tubulin expression, and 70% showed a strong staining intensity. The high and persistent expression of Sox2 and βIII-tubulin in PDC support their common origin on a neural progenitor.

The limitations of our study include its small sample size and retrospective nature. The limited number of patients and missing follow-up information may have affected the significance of our results. We analyzed all histological subtypes together due to the small number of individual cases with follow-up data. Thus, it remains to be seen whether the value of βIII-tubulin and Sox2 expression in PDC can be validated in a prospective study. 

## 5. Conclusions

The remarkable high prevalence of Sox2 and βIII-tubulin expression in PDC emphasizes the putative relevance of these markers in these tumors. The absence of significant differences in the expression of these markers among different subtypes of PDC supports the hypothesis of their common origin from a neural stem/progenitor cell. Sox2 overexpression correlated with longer DFS and less distant metastasis, while high βIII-tubulin expression also correlated with longer DFS. More studies evaluating these markers in PDC should be carried out to further determine their clinical potential.

## Figures and Tables

**Table 1:** Clinical data of patients according to tumor histology.

	SNUC	SNEC	ONB
**Total Patients**	36	8	13
**Gender**			
Male	19	3	4
Female	17	5	9
**Age**			
Average	57	58	47
Range	31–85	40–77	20–69
**Location**			
Maxillary sinus	6	0	0
Ethmoid sinus	30	8	13
**T classification**			
T2	14	4	6
T3	2	1	2
T4a	19	2	5
T4b	1	1	0
**Disease stage**			
I-II	14	4	6
III-IV	32	4	7
**Radiotherapy**			
No	3	0	0
Yes	33	8	13
**Follow-up**			
Mean	29	22	56
Median	13	25	29
Range	1–97	15–26	1–172
**Local Recurrence**			
No	11	1	4
Yes	5	2	3
**Distant metastasis**			
No	11	0	4
Yes	5	3	3
**Patient status**			
Alive	8	1	6
Died of disease	6	2	1
Died of other causes	2	0	0
Lost	20	5	6

**Table 2:** Diagnostic immunohistochemical staining results.

	SNUC	SNEC	ONB
	*n* = 36	*n* = 8	*n* = 13
**Pancytokeratin**	97%	100%	38%
**CK 5/6**	0%	ND	ND
**P40**	0%	ND	ND
**P16**	27%	0%	0%
**HPV**	0%	0%	0%
**Synaptophysin/Chromogranin**	39%	100%	100%
**NUT**	3%	0%	0%
**SMARCB1 (INI-1)**	3%	0%	0%
**SMARCA4 (BRG1)**	0%	12.5%	0%
**IDH2 mut**	28%	25%	0%

**Table 3:** Sox2 and βIII-tubulin staining results.

	SNUC	SNEC	ONB
	*n* = 36	*n* = 8	*n* = 13
**Sox2** nuclear	53%	75%	46%
**βIII-tubulin** strong	64%	75%	85%
moderate	8%	12.5%	8%
weak	17%	12.5%	0%
negative	11%	0%	0%

## Data Availability

All the data are available in the text.
